# Spinal poly-GA inclusions in a *C9orf72* mouse model trigger motor deficits and inflammation without neuron loss

**DOI:** 10.1007/s00401-017-1711-0

**Published:** 2017-04-13

**Authors:** Martin H. Schludi, Lore Becker, Lillian Garrett, Tania F. Gendron, Qihui Zhou, Franziska Schreiber, Bastian Popper, Leda Dimou, Tim M. Strom, Juliane Winkelmann, Anne von Thaden, Kristin Rentzsch, Stephanie May, Meike Michaelsen, Benjamin M. Schwenk, Jing Tan, Benedikt Schoser, Marianne Dieterich, Leonard Petrucelli, Sabine M. Hölter, Wolfgang Wurst, Helmut Fuchs, Valerie Gailus-Durner, Martin Hrabe de Angelis, Thomas Klopstock, Thomas Arzberger, Dieter Edbauer

**Affiliations:** 1 German Center for Neurodegenerative Diseases (DZNE) Munich, Feodor-Lynen-Straße 17, 81377 Munich, Germany; 2 Munich Cluster for System Neurology (SyNergy), Feodor-Lynen-Straße 17, 81377 Munich, Germany; 3 German Mouse Clinic, Institute of Experimental Genetics, German Research Center for Environmental Health, Helmholtz Zentrum München, Ingolstädter Landstrasse 1, 85764 Neuherberg, Germany; 4 Institute of Developmental Genetics, German Research Center for Environmental Health, Helmholtz Zentrum München, Ingolstädter Landstrasse 1, 85764 Neuherberg, Germany; 5 Department of Neuroscience, Mayo Clinic, 4500 San Pablo Road, Jacksonville, FL 32224 USA; 6 Department of Anatomy and Cell Biology, Biomedical Center, Ludwig-Maximilians-University Munich, Großhaderner Str. 9, 82152 Planegg-Martinsried, Germany; 7 Physiological Genomics, Biomedical Center, Ludwig-Maximilians-University Munich, Großhaderner Str. 9, 82152 Planegg-Martinsried, Germany; 8 Molecular and Translational Neuroscience, Department of Neurology, University of Ulm, Albert-Einstein-Allee 11, 89081 Ulm, Germany; 9 Institut für Humangenetik, Helmholtz Zentrum München, 85764 Munich, Germany; 10 Institut für Neurogenomik, Helmholtz Zentrum München, 85764 Munich, Germany; 11 Neurologische Klinik, Klinikum rechts der Isar, Technische Universität München, 81675 Munich, Germany; 12 Institut für Humangenetik, Klinikum rechts der Isar, Technische Universität München, 81675 Munich, Germany; 13 Department of Neurology, Friedrich-Baur-Institute, Klinikum der Ludwig-Maximilians-Universität München, Ziemssenstr. 1a, 80336 Munich, Germany; 14 Chair of Developmental Genetics, Technische Universität München, Freising-Weihenstephan, Germany; 15 Chair of Experimental Genetics, School of Life Science Weihenstephan, Technische Universität München, Alte Akademie 8, 85354 Freising, Germany; 16 German Center for Diabetes Research (DZD), Ingolstädter Landstr. 1, 85764 Neuherberg, Germany; 17 Center for Neuopathology and Prion Research, Ludwig-Maximilians-University Munich, Feodor-Lynen-Straße 23, 81377 Munich, Germany; 18 Department of Psychiatry and Psychotherapy, Ludwig-Maximilians University Munich, Nußbaumstraße 7, 80336 Munich, Germany; 19 Institute for Metabolic Biochemistry, Ludwig-Maximilians-University Munich, Feodor-Lynen-Straße 17, 81337 Munich, Germany

**Keywords:** ALS, FTLD, FTD, MND, C9orf72, Neurodegeneration, Neurological disorder, Mouse model

## Abstract

Translation of the expanded (ggggcc)_n_ repeat in *C9orf72* patients with amyotrophic lateral sclerosis (ALS) and frontotemporal dementia (FTD) causes abundant poly-GA inclusions. To elucidate their role in pathogenesis, we generated transgenic mice expressing codon-modified (GA)_149_ conjugated with cyan fluorescent protein (CFP). Transgenic mice progressively developed poly-GA inclusions predominantly in motoneurons and interneurons of the spinal cord and brain stem and in deep cerebellar nuclei. Poly-GA co-aggregated with p62, Rad23b and the newly identified Mlf2, in both mouse and patient samples. Consistent with the expression pattern, 4-month-old transgenic mice showed abnormal gait and progressive balance impairment, but showed normal hippocampus-dependent learning and memory. Apart from microglia activation we detected phosphorylated TDP-43 but no neuronal loss. Thus, poly-GA triggers behavioral deficits through inflammation and protein sequestration that likely contribute to the prodromal symptoms and disease progression of *C9orf72* patients.

**Electronic supplementary material:**

The online version of this article (doi:10.1007/s00401-017-1711-0) contains supplementary material, which is available to authorized users.

## Introduction

A (ggggcc)_n_ hexanucleotide repeat expansion upstream of the coding region of *C9orf72* is the most common genetic cause of amyotrophic lateral sclerosis (ALS) and frontotemporal dementia (FTD), with some patients showing symptoms of both diseases [7]. Patients usually carry several hundred or thousand ggggcc repeats compared to less than 30 in the general population. The repeat expansion inhibits *C9orf72* expression, and sense and antisense transcripts may cause toxicity by sequestering RNA-binding proteins in nuclear foci [6, 28]. Moreover, both sense and antisense repeat transcripts are translated from all reading frames into aggregating dipeptide repeat (DPR) proteins (poly-GA, -GP, -GR, -PA, and -PR) [1, 22, 23, 40]. The relative contribution of these putative pathomechanisms, and their link to the co-occurring TDP-43 pathology present in patients with *C9orf72* ALS/FTD, are under intense debate.

Generating mouse models for *C9orf72* repeat expansion diseases has been surprisingly challenging [13]. Complete loss of *C9orf72* does not cause neurodegeneration, but does affect autophagy, particularly in the immune system, and leads to splenomegaly [15, 25]. High level viral expression of a relatively short (ggggcc)_66_ repeat expansion leads to rapid neurodegeneration accompanied by DPR and TDP-43 pathology [5]. In contrast, expressing lower levels of an expanded repeat in its endogenous context leads to variable results. Two BAC transgenic mouse lines showed the characteristic RNA foci and DPR inclusions of *C9orf72* ALS/FTD, but no neuron loss or behavioral symptoms [24, 26], while two similar mouse models additionally showed cognitive symptoms [15, 19]. The more dramatic effects in the viral system may be due to higher expression levels and altered processing of exonic repeat expression [34]. Together, these models strongly support gain of function toxicity as the main cause of *C9orf72* ALS/FTD, but cannot differentiate the contribution of sense and antisense RNA transcripts and the five DPR species. Viral expression of the most abundant DPR species, poly-GA, in the mouse brain causes mild neurodegeneration and cognitive symptoms without TDP-43 pathology, but this system showed no expression in the spinal cord [37]. In patients, DPR proteins are less common in the spinal cord than in the brain, but they are also found in upper and lower motoneurons [31].

To elucidate the specific contribution of poly-GA to disease pathogenesis, we aimed to generate a transgenic mouse model with poly-GA expression levels comparable to *C9orf72* ALS/FTD patients. We chose a *Thy1*-based expression vector for neuron-specific expression of poly-GA [9]. Here, we report in-depth pathological and phenotypic analyses of these mice focusing on the motor system.

## Methods

### Generation of *Thy1* (GA)_149_-CFP mice

We inserted a multiple cloning site into the pUC18 based murine Thy1.2 vector using synthetic oligonucleotides [9]. This allowed us to insert a cDNA encoding (GA)_149_, 31 amino acids corresponding to the 3′ region of the poly-GA reading frame in patients [22] and cyan fluorescent protein (CFP) (sequence shown in Fig. S1a). Compared to the previous (GA)_149_-GFP construct [21] only the fluorescent protein had been changed. Linearized vector was injected into C57BL/6-derived zygotes and transferred into pseudopregnant CD1 females (PolyGene). GA-CFP mice were kept in the C57BL/6N background. Mice were PCR genotyped using the following primers (tccaggagcgtaccatcttc; gtgctcaggtagtggttgtc). We confirmed maintenance of the full length transgene with PCR amplification (Expand Long Template PCR System, Roche, 11681842001; gatccaagcttgccaccatg; tctagctctgccactccaag) and sequencing.

The transgene integration site was determined by whole genome sequencing according to standard protocols using the TruSeq DNA PCR-Free Library Preparation Kit and an Illumina HiSeq 4000 with 150 bp paired-end reads resulting in about 58× coverage from two lanes. Sequences mates mapping to different chromosomes were analyzed using the Integrative Genomics Viewer (IGV) [33].

### Immunohistochemistry of mouse and patient tissue

After killing, 1-, 3-, 6-, and 12-month-old mice were transcardially perfused with 1% sterile PBS and tissue was then formalin fixated for 2 days. Histological stainings were performed on 5–8 µm thick sections from paraffin-embedded tissue. For spinal cord tissue, an additional decalcification step with 5% formic acid for 5 days was performed after formalin fixation. After deparaffinization in xylene and dehydration in graded ethanol, the paraffin sections were treated with citrate buffer (pH 6) for 20 min in the microwave. Mlf2 IHC staining was more prominent when the citrate retreatment was followed by 20 min incubation in 80% formic acid or 5–25 min incubation with 0.1 µg/µl proteinase K in 10 mM Tris/HCl pH 7.6 at 37 °C. Afterwards the slides were incubated with primary antibody overnight at 4 °C. For ChAT, an additional incubation with rabbit anti-goat-IgG was performed the next day for 1 h at room temperature. The slides were detected by the DCS SuperVision 2 Kit (DCS innovative diagnostic-system, Hamburg, Germany) according to the manufacturer’s instructions. Iba1 and GFAP immunohistochemistry was performed with the Ventana BenchMark XT automated staining system (Ventana) using the UltraView Universal DAB Detection Kit (Roche). For Nissl staining, the deparaffinized slides were incubated in 70% ethanol overnight. After 30 min in Cresyl violet and 1 min in 96% ethanol the slides were processed in 100% ethanol with glacial acetic acid. Bright-field images were taken by CellD, Olympus BX50 Soft Imaging System (Olympus, Tokyo, Japan).

For immunofluorescence, after deparaffinization and citrate antigen retrieval, the slides were incubated with primary antibody overnight at 4 °C and the following day incubated for 1 h at room temperature with secondary Alexa Fluor labeled antibodies. For Mlf2 immunofluorescence staining, a 1 min treatment at 37 °C with 0.05 µg/µl proteinase K in 10 mM Tris/HCl pH 7.6 was necessary before citrate antigen retrieval. After the nuclei were counterstained with DAPI, the slices were incubated for 1 min in 0.2% Sudan black B and mounted with Fluoromount Aqueous Mounting Medium (Sigma, F4680). Fluorescent images were taken using a LSM710 confocal laser scanning system (Carl Zeiss, Jena, Germany) with 20x or 40x/63x oil immersion objectives.

### Antibodies

α-GFP (1:1000, Clonetech 632592), α-GA clone 5F2 [20] (purified mouse monoclonal, WB unlabeled 1:50; IHC HRP labeled 5F2 1:2500, labeled by AbD Serotec HRP-labeling Kit LNK002P; biotinylated 5F2 7 ng/µl; MSD-labeled 5F2 10 ng/µl, labeled by Meso Scale MSD Sulfo-Tag NHS-Ester R91AN-1), α-GA-CT (C-terminal tail) clone 5C3 [22] (rat monoclonal, 1:50), α-p62/SQSTM1 (IF 1:100, IHC 1:1000, MBL, PM045), α-pTDP-43 (Ser409/Ser410) clone 1D3 [20] (purified rat monoclonal, 1:50), α-TDP-43 (1:1000, Cosmo Bio, TIP-TD-P09), α-RanGAP1 (1:100, Abcam, ab92360), α-nucleolin (1:1000, Abcam, ab50729), α-CD68 (1:1000, Abcam, ab125212), α-Iba1 (1:500, Wako, 091-19741), α-GFAP (1:5000,Dako, Z0334), α-NeuN (1:1000, Abcam, ab177487), α-ChAT (IF 1:300, IHC 1:5000, Millipore, AB144P), α-Calnexin (1:3000, Enzo Life Science, SPA-860F), α-Calbindin (1:300, Abcam, ab49899), α-Calretinin (1:1000, Abcam, ab702) α-Parvalbumin (1:750, Abcam, ab11427), α-Mlf2 #1 (1:1000, Sigma-Aldrich, HPA010811-100UL), α-Mlf2 #2 (1:1000, Santa Cruz, sc-166874), α-Laminin (1:200, Abcam, ab11575), α-goat-IgG (1:400, Dako, E0466), α-mouse Alexa Fluor 488 (1:500, Thermo Fischer Scientific, A11029), α-rabbit Alexa Fluor 488 (1:500, Thermo Fischer Scientific, A11034), α-rat Alexa Fluor 488 (1:500, Thermo Fischer Scientific, A11006), α-mouse Alexa Fluor 555 (1:500, Thermo Fischer Scientific, A21424), α-rabbit Alexa Fluor 555 (1:500, Thermo Fischer Scientific, A21429), α-rat Alexa Fluor 555 (1:500, Thermo Fischer Scientific, A21434), Streptavidin Alexa Fluor 488 (1:500, Thermo Fischer Scientific, S11223), nuclei were stained with DAPI (Roche Applied Science, Penzberg, Germany).

### Immunoassay analysis of poly-GA in tissue homogenates

Mouse brainstem and spinal cord samples and *C9orf72* patient motor cortex samples were sonicated in 500–700 µl of cold RIPA buffer (137 mM NaCl, 20 mM Tris pH 7.5, 10% Glycin, 1% Triton X 100, 0.5% Na-deoxycholate, 0.1% SDS, 2 mM EDTA, protease and phosphatase inhibitors). 100 µl of this homogenized tissue stock solutions were diluted to 300 µl with RIPA and centrifuged at 100,000×*g* for 30 min at 4 °C. To avoid cross contamination, the RIPA-insoluble pellets were resuspended in 300 µl RIPA, re-sonicated and re-centrifuged. Afterwards the RIPA-insoluble pellets were sonicated in U-RIPA (RIPA buffer containing 3.5 M Urea) and the protein concentration determined by Bradford assay. Streptavidin Gold multi-array 96-well plates (Mesoscale, L15SA-1) were blocked for 30 min with block solution (1% BSA, 0.05% Tween20 in PBS) and incubated with biotinylated α-GA clone 5F2 overnight at 4 °C. Equal amounts of protein of all samples were added in duplicate wells for 2 h, followed by 2 h incubation with the secondary MSD-labeled α-GA clone 5F2. Serial dilution of recombinant GST-GA_15_ in blocking buffer was used to prepare a standard curve. The wells intensity of emitted light upon electrochemical stimulation was measured using the MSD Quickplex 520 and the background corrected by the average response obtained from blank wells. Sensitivity and specificity of the immunoassay were confirmed using purified 15-mer DPRs fused to GST (Fig. S3a, b).

### Phenotypic analysis of mice

The study was conducted in accordance with European and national guidelines for the use of experimental animals, and the protocols were approved by the governmental committee (Regierungspräsidium Oberbayern, Germany). All experimenters were blind to the genotype.

Barnes maze (Stoelting Europe, Ireland) assay to test spatial, hippocampus-dependent long-term memory in mice was performed on a circular surface (diameter 91 cm) with 20 circular holes (diameter 5 cm) around its circumference [3]. Under one hole was an “escape box” (diameter 4 cm, depth 15 cm). The table surface was brightly lit by overhead lightning (900 lx). For each trial the mice had 3 min to find and hide in the “escape box”. For the statistical analysis failed attempts were set to 3 min.

In the balance beam test, the mice were placed on a wooden beam (round surface, length 58 cm, diameter 8 mm) and had 1 min to cross the beam. The test was finished either when the mice reached the end of the stick, they dropped down or the time ran out. For the statistical analysis failed attempts were set to 1 min. The experimenters were blind to the genotype, and trials were either video documented or recorded by AnyMaze (Stoelting Europe). AnyMaze Software was used to track the mice and to analyze the data.

In the Rotarod test (Ugo Basile), we accelerated the spindle speed from 5 to 50 rpm over 5 min. The test finished either after 5 min or when the mouse dropped down. The average time of two trials with 1 h break in between was used.

Modified SHIRPA analysis and grip strength testing was performed as described [11].

The beam ladder consists of two Plexiglas screens connected with several metal beams of variable distance. The test is used to evaluate skilled walking of the mice. Mice traverse the ladder and foot slips of fore paws and hind paws are counted separately as well as the time to traverse the beam.

The open field test as an assessment of spontaneous exploratory and anxiety-related behavior in a novel environment was carried out as previously described [12, 14, 39]. It consisted of a transparent and infra-red light permeable acrylic test arena with a smooth floor (internal measurements: 45.5 × 45.5 × 39.5 cm). Illumination levels were set at approximately 150 lx in the corners and 200 lx in the middle of the test arena. Each animal was placed individually into the middle of one side of the arena facing the wall and allowed to explore it freely for 20 min. For data analysis, the arena was divided by the computer in two areas, the periphery defined as a corridor of 8 cm width along the walls and the remaining area representing the center of the arena (42% of the total arena). Data were recorded and analyzed using the ActiMot system (TSE, Bad Homburg, Germany).

Acoustic startle and its prepulse inhibition were assessed using a startle apparatus setup (Med Associates Inc., VT, USA) including four identical sound-attenuating cubicles. The protocol is based on the Eumorphia protocol (http://www.empress.har.mrc.ac.uk), adapted to the specifications of our startle equipment, and constantly used in the primary screen of the GMC [30]. Background noise was 65 dB, and startle pulses were bursts of white noise (40 ms). A session was initiated with a 5-min-acclimation period followed by five presentations of leader startle pulses (110 dB) that were excluded from statistical analysis. Trial types included prepulse alone trials at four different sound pressure levels (67, 69, 73, 81 dB), and trials in which each prepulse preceded the startle pulse (110 dB) by a 50 ms inter-stimulus interval. Each trial type was presented ten times in random order, organized in ten blocks, each trial type occurring once per block. Inter-trial intervals varied from 20 to 30 s.

### DNA constructs and lentivirus production

cDNA of rat Mlf2 (NCBI Gene ID: 312709) containing an N-terminal HA-tag was expressed from a lentiviral vector driven by human ubiquitin promoter (FUW2-HA). Previously described (GA)_175_-GFP cDNA expressed from a synthetic gene lacking repetitive (ggggcc)_n_ sequences with ATG start codon and EGFP was cloned in a lentiviral packing vector (FhSynW2) containing the human synapsin promoter [21]. Lentivirus was produced in HEK293FT cells (Life Technologies) as described previously [10].

### Cell culture, RNA isolation and immunoprecipitation

Primary hippocampal neurons from embryonic day 19 rats were cultured and transduced with lentivirus as described previously [32]. Immunofluorescence staining was performed on 10 min PFA (4% paraformaldehyde and 4% sucrose) fixed primary neurons. The primary and secondary antibodies were diluted in GDB buffer (0.1% gelatin, 0.3% Triton X-100, 450 mM NaCl, 16 mM sodium phosphate pH 7.4) and incubated over night at 4 °C or 1 h at room temperature. Confocal images were taken using a LSM710 confocal laser scanning system (Carl Zeiss, Jena, Germany) with 40× or 63× oil immersion objectives. RNA isolation and qPCR was performed as described previously [23] using the following primers (CD68 ttctgctgtggaaatgcaag and gagaaacatggcccgaagt; Iba1 acagcaatgatgaggatctgc and ctctaggtgggtcttgggaac; GFAP tttctcggatctggaggttg and agatcgccacctacaggaaa; ACTB atggaggggaatacagccc and ttctttgcagctccttcgtt; GAPDH caacagcaactcccactcttc and ggtccagggtttcttactcctt).

### Patient material

Tissue samples of patient autopsy cases were provided by the Neurobiobank Munich, Ludwig-Maximilians-University (LMU) Munich and collected according to the guidelines of the local ethics committee.

### Statistics and analysis

Statistical analysis was performed with GraphPad Prism software (version 7.01). For neuron and motoneuron count, images of the left and right anterior horns of the spinal cord were taken and all positively stained cells were manually counted. The count number represents the neurons/motoneurons averaged on one side. Experiments with two groups were analyzed by *t* test (unpaired, two-sided, *t* = size of the difference relative to the variation; *df* = degrees of freedom). Behavioral data was analyzed by two-way ANOVA with Bonferroni post hoc test (*F* = equality of variances).

### Phospho-TDP-43 immunoassays

For phosphorylated TDP-43 measurements, sarkosyl-soluble and urea-soluble fractions of mouse spinal cord tissues were prepared as previously described [5]. In brief, 25–60 mg of tissue were subjected to a sequential extraction protocol using Tris–EDTA buffer (50 mM Tris pH 7.4, 50 mM NaCl, 1 mM EDTA), high salt Triton X-100 buffer, Triton X-100 buffer + 30% sucrose, and sarkosyl buffer. Sarkosyl-insoluble material was further extracted in urea buffer. The protein concentrations of sarkosyl-soluble fractions were determined using a bicinchoninic acid assay (Thermo Scientific), whereas a Bradford assay was utilized to measure protein concentrations of urea-soluble fractions. Phosphorylated TDP-43 levels in both these fractions were evaluated using a sandwich immunoassay that utilizes MSD electrochemiluminescence detection technology [15]. A mouse monoclonal antibody that detects TDP-43 phosphorylated at serines 409 and 410 (Cosmo Bio, #CAC-TIP-PTD-M01, 1:500) was used as the capture antibody. The detection antibody was a sulfo-tagged rabbit polyclonal C-terminal TDP-43 antibody (Proteintech, 12892-1-AP, 2 µg/ml). Response values corresponding to the intensity of emitted light upon electrochemical stimulation of the assay plate using the MSD QUICKPLEX SQ120 were acquired and background corrected using the average response from buffer only.

## Results

### *Thy1* (GA)_149_-CFP mice accumulate poly-GA inclusions in the spinal cord and brainstem

**Fig. 1:**
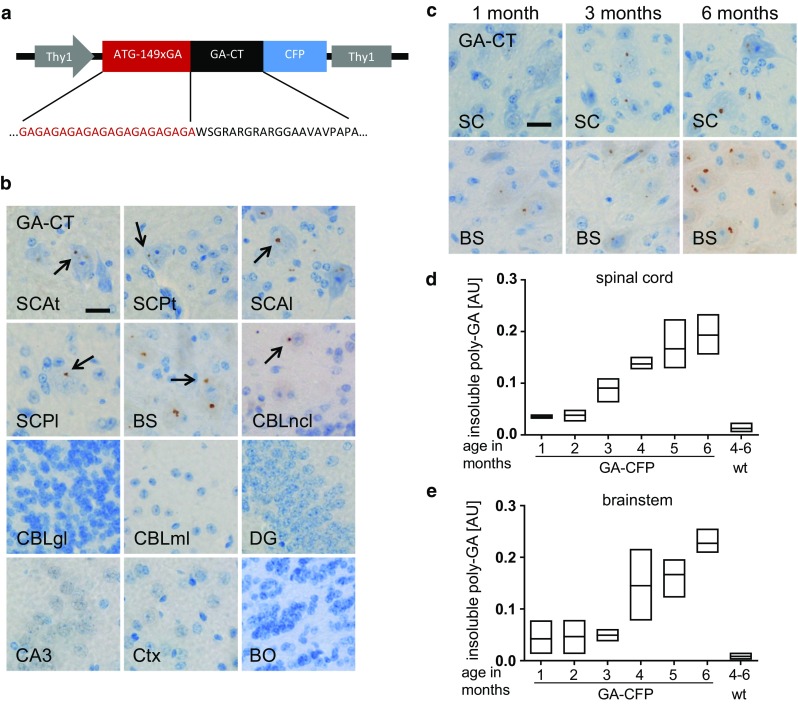
Expression and distribution pattern of poly-GA aggregates in GA-CFP mice. **a** Schematic diagram of the construct containing the murine Thy1 promoter driving expression of a synthetic gene encoding (GA)_149_ with its endogenous C-terminal tail fused to CFP. (GA)_149_-CFP is replacing the endogenous coding region. **b** Distribution of GA aggregates show many inclusions in the spinal cord and brainstem and no aggregates in cortical regions, hippocampus or molecular and granular layer of the cerebellum. *BO* olfactory bulb, *BS* brainstem, *CA3* cornu ammonis fields 3, *CBLgl* cerebellar granular cell layer, *CBLml* cerebellar molecular cell layer, *CBLncl* lateral cerebellar nuclei, *DG* dentate gyrus, *SCAl* anterior horn of lumbar spinal cord, *SCAt* anterior horn of thoracic spinal cord, *SCPl* posterior horn of lumbar spinal cord, *SCPt* posterior horn of thoracic spinal corn. *Scale bars* represent 20 µm. **c** Increasing number and accumulation of aggregates in spinal cord (SC; *upper row*) and brainstem (BS; *lower row*) of 1-, 3- and 6-month-old GA-CFP mice detected by immunohistochemical staining using GA-CT antibody. *Scale bar* represents 20 µm. Quantitative immunoassay of RIPA-insoluble poly-GA in the spinal cord (**d**) and brainstem (**e**) of 1–6-month-old GA-CFP mice (*n* = 3 mice per time-point; measured in duplicates) shows increasing amounts of poly-GA in a time dependent manner. *AU* arbitrary unit, data are shown as mean, minimum and maximum

We generated a *Thy1*-based vector to express (GA)_149_ using a synthetic sequence, which unlike the repeat expansion in patients has no extensive (ggggcc)_n_ stretches, fused with a C-terminal fluorescent CFP tag (Figs. 1a, S1a). Since the relevance of the C-terminal tail of endogenous DPR products is unknown, we additionally included 31 amino acids translated from the endogenous locus in the poly-GA reading frame [22]. Using pronuclear injections into C57BL/6 mice, we generated a founder line (termed GA-CFP) with germline transmission and poly-GA expression. Transgenic mice were born at Mendelian frequency and did not differ in adult viability. Sequencing confirmed transmission of the full length open reading frame in all analyzed animals (*n* = 3, data not shown) and genomic PCR from different tissues further confirmed the somatic stability of the synthetic repeat gene (Fig. S1b). We identified the integration site using whole genome sequencing and validated our findings by PCR and Sanger sequencing (Fig. S2). Several transgene copies integrated on chromosome 14 about 330 kb downstream of the nearest transcript, the long non-coding RNA 4930474H20Rik, strongly suggesting that no endogenous genes are disrupted.

Using immunohistochemistry, we characterized poly-GA protein expression in different brain regions in 4–6-month-old mice. Expression of the aggregated full length product was detected with antibodies targeting CFP, poly-GA or the C-terminal DPR tail (GA-CT) (Fig. S1c). While most of the poly-GA inclusions were cytoplasmic, a few inclusions were observed in the nucleus (Fig. S1d). In GA-CFP mice, poly-GA-inclusion pathology was restricted to neurons of brain stem, cerebellar nuclei and spinal cord. There were numerous poly-GA-immunopositive inclusions in large neurons of the brainstem, the lateral (dentate) and interposed cerebellar nuclei and (most abundantly) the anterior horn of the spinal cord, particularly in the cervical, thoracic and lumbar regions (Fig. 1b). Inclusion pathology was additionally observed in interneurons (Fig. S1e) in the laminae IV, V and VI of the posterior horn. No poly-GA inclusions were detected in the olfactory bulb, the molecular and granular layer of the cerebellum, the hippocampus or the neocortex, including the motor cortex (Fig. 1b).

Next, we analyzed the progression of poly-GA pathology in GA-CFP mice with age. Inclusions were visible by IHC in the spinal cord and brain stem at 1 month of age, and the number and size of inclusions increased with age (Fig. 1c). Consistent with these findings, levels of RIPA-insoluble poly-GA in brain stem and spinal cord lysates increased over time, as assessed using a poly-GA-specific immunoassay (Figs. 1d, e, S3a, S3b), whereas no signal was detected in non-transgenic littermates. No poly-GA was detectable in the RIPA-soluble fraction of the spinal cord and brainstem (Fig. S3c). Fair comparison of poly-GA levels in mice and patients is complicated by the different regional expression pattern in mice and patients and variable poly-GA levels in patients. However, we measured the expression of poly-GA in spinal cord of 4–6-month-old GA-CFP mice and motor cortex of *C9orf72* ALS/FTD patients with abundant poly-GA pathology by immunoassay (Fig. S3d) and additionally counted the frequency of neuronal poly-GA inclusions in the most affected regions of GA-CFP mice and the neocortex of *C9orf72* patients (Fig. S3e, f). Both assays show that poly-GA expression is not grossly exaggerated in GA-CFP mice. Thus, GA-CFP mice are a suitable model to address the pathomechanisms of poly-GA in the motor system.

### Poly-GA co-aggregates with p62, Rad23b and the chaperone-associated protein Mlf2

**Fig. 2:**
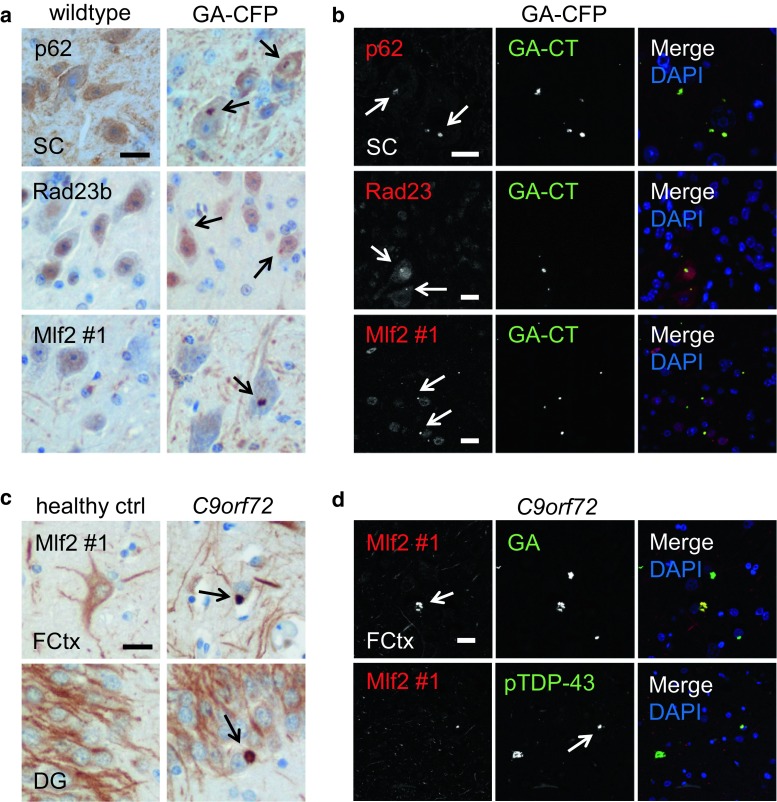
GA-CFP mice develop p62, Rad23b and Mlf2 pathology similar to human *C9orf72* mutation carriers. **a** Immunohistochemistry shows p62, Rad23b and Mlf2 aggregates in the spinal cord (SC) of 6-month-old GA-CFP mice but not of wildtype mice. **b** Immunofluorescence stainings show p62, Rad23b and Mlf2 positive inclusions that co-localize with poly-GA in the spinal cord of 6-month-old GA-CFP mice. **c** Immunohistochemistry detects specific Mlf2 aggregates in the frontal cortex (FCtx) and dentate gyrus (DG) of *C9orf72* ALS/FTLD patients. **d** Double immunofluorescence reveals colocalization of Mlf2 aggregates with poly-GA and phosphorylated TDP-43 inclusions in *C9orf72* patients. *Scale bars* represent 20 µm

To investigate potential downstream effects of poly-GA expression, we analyzed whether poly-GA co-aggregates with other proteins. Similar to findings in *C9orf72* ALS/FTD patients [23], the vast majority of poly-GA inclusions co-localized with p62 (Fig. 2a, b, first row and Table S1). Rad23b, a known poly-GA-interacting protein involved in the ubiquitin proteasome pathway, also aggregated in GA-CFP mice (Fig. 2a, b, second row) similar to previous reports [21, 37]. In contrast to overexpression of poly-GA in rat primary neurons [21], GA-CFP mice showed no sequestration of Unc119 (Fig. S4a) and no mislocalization or co-localization of RanGAP1 with poly-GA (Fig. S4b first row, and Table S1), which is consistent with our cell culture data [17]. We also found no evidence of nucleolar pathology using nucleolin immunostaining (Fig. S4b second row).

Additionally, we analyzed whether poly-GA co-aggregates with proteins identified in poly-GA immunoprecipitates in primary hippocampal neurons in our recent mass spectrometry screen [21]. Among such proteins that had not been previously validated, the Hsp70-associated protein, Mlf2, showed the strongest co-aggregation with poly-GA in the spinal cord of GA-CFP mice, whereas no Mlf2 aggregates were detected in wildtype mice (Fig. 2a, b, third row). Co-transduction of HA-Mlf2 and (GA)_175_-GFP in primary hippocampal neurons of wildtype rats corroborated the sequestration of Mlf2 into poly-GA inclusions (Fig. S4c). Moreover, endogenous Mlf2 was sequestered into poly-GA inclusions in primary neurons transduced with (GA)_175_-GFP (Fig. S4d). These data led us to examine Mlf2 aggregation in *C9orf72* ALS/FTD patients. We detected Mlf2 pathology in the frontal cortex and hippocampus of *C9orf72* ALS/FTD patients but not healthy controls using two independent Mlf2 antibodies (Figs. 2c, S4e). In addition, double immunofluorescence staining confirmed the co-aggregation of Mlf2 with poly-GA in *C9orf72* patients (Fig. 2d, first row). While in GA-CFP mice Mlf2 was co-aggregating in ~55% of the poly-GA inclusions, in *C9orf72* patients only 0.3–2.7% of the poly-GA aggregates showed Mlf2 sequestration, depending on the brain region (Table S2). However, we detected Mlf2 also occasionally in cytoplasmic phospho-TDP-43 inclusions in *C9orf72* patients (Fig. 2d, second row). Thus, our GA-CFP mice recapitulate the poly-GA component of pathology in *C9orf72* ALS/FTD patients, including the co-aggregation of poly-GA with p62, Rad23b and Mlf2.

### Poly-GA triggers mild TDP-43 phosphorylation but no overt neuron loss

**Fig. 3:**
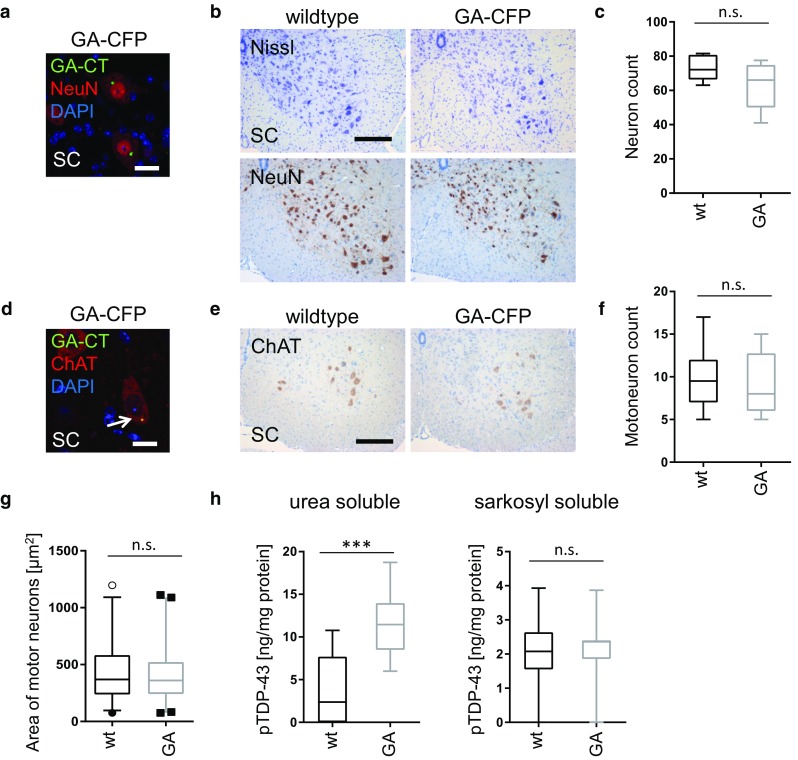
GA-CFP mice show no evidence for neuronal loss but increased TDP-43 phosphorylation. **a** Double immunofluorescence of 6-month-old GA-CFP spinal cord tissue (SC) shows poly-GA inclusions exclusively in NeuN-positive cells. *Scale bar* represents 20 µm. **b**, **c** Nissl staining and NeuN immunohistochemistry of 6-month-old GA-CFP and wildtype spinal cords. *Scale bar* represents 100 µm. Quantitative analysis of NeuN-positive neurons shows no significant difference between wildtype and GA-CFP mice (*n*_GA-CFP/wt_ = 3). **d** Immunostaining of poly-GA aggregates in choline acetyltransferase (ChAT)-positive motoneurons. *Scale bar* represents 20 µm. **e**–**g** Immunohistochemistry and quantitative analysis of ChAT-positive motoneurons of 6-month-old mice in the anterior horn of the spinal cord revealed no statistically significant differences in neuron count (*n*_GA-CFP/wt mice_ = 4) and size (*n*_GA-CFP motoneurons_ = 228; *n*_wt motoneurons_ = 195). Neurons were counted as described in the “Statistics” section. *Scale bar* represents 100 µm. **h** Immunoassay for phosphorylated TDP-43 in sarkosyl (1%)-soluble or urea (7M)-soluble spinal cord fractions from 6-month-old GA-CFP or wildtype (wt) mice. *n*_(wt)_ = 12; *n*_(GA-CFP)_ = 8. Unpaired *t* test (two-tailed; sarkosyl *t* = 0.3034, *df* = 18; urea *t* = 4.172, *df* = 18). Data are shown as box plot with whiskers at the 1st and 99th percentile. ***p < 0.001, *ns* not significant

We next analyzed whether poly-GA expression drives neurodegeneration. Consistent with the expression pattern of the *Thy1* promoter, poly-GA was exclusively found in NeuN-positive neurons (Fig. 3a) and no expression was detectable in microglia or muscle fibers (Fig. S4f). However, Nissl staining and NeuN immunostaining revealed no overt neuron loss in the spinal cord (Fig. 3b, c). Poly-GA was found in most choline acetyltransferase (ChAT) positive motoneurons in the anterior horn of the spinal cord (Fig. 3d), but ChAT immunostaining revealed no statistically significant loss of motoneurons at 6 months (Fig. 3e–g). Furthermore, the size and shape of motoneurons in GA-CFP mice did not show signs of degeneration and were not discernible from the corresponding neurons in wildtype mice.

Next, we analyzed another neuropathological hallmark of ALS, namely TDP-43 phosphorylation, in aged GA-CFP mice. We quantified levels of phosphorylated TDP-43 (at serines 409 and 410) in the spinal cord of mice at 6 months of age by ELISA. Phosphorylated TDP-43 levels were approximately threefold higher in the urea-soluble (but sarkosyl-insoluble) fraction of GA-CFP mice compared to wildtype mice, but no difference was detected in the sarkosyl-soluble fraction (Fig. 3h). While mature TDP-43 inclusions and cytoplasmic TDP-43 mislocalization were not observed in GA-CFP mice, even at 12 months of age (Fig. S4g, h; Table S1), these data may nonetheless indicate that poly-GA contributes to the onset of TDP-43 pathology.

### Poly-GA induces microglia activation without astrogliosis

**Fig. 4:**
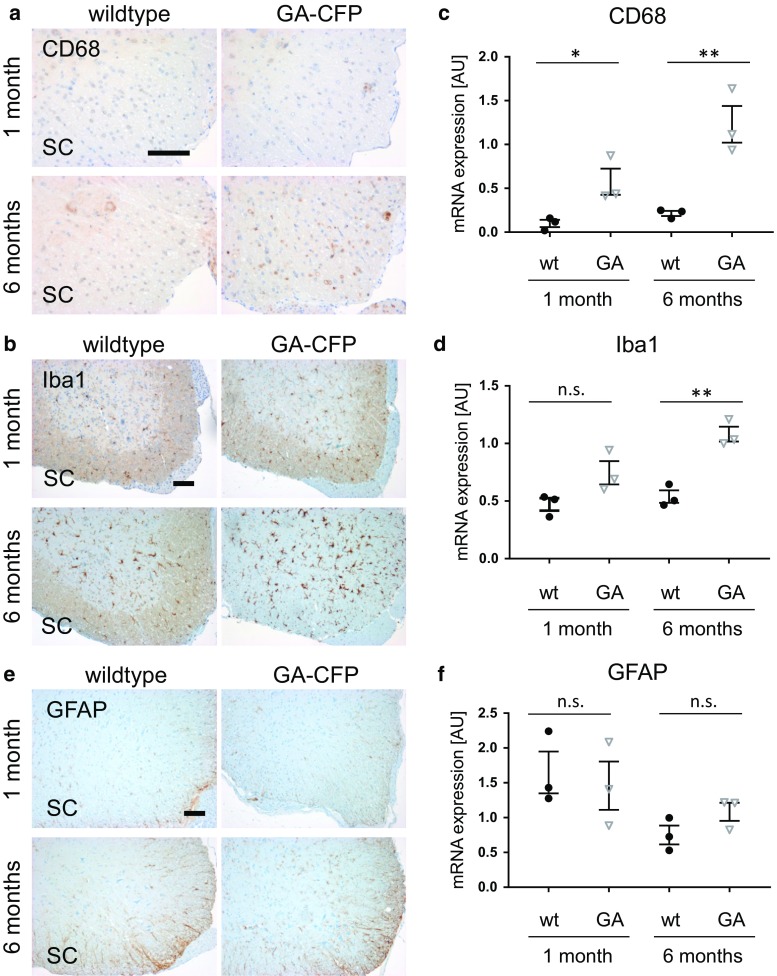
Activation of phagocytic microglia in GA-CFP mice. **a**, **b**, **e** Immunohistochemistry of 1- and 6-month-old mice shows microglia activation detected by the markers CD68 and Iba1 in the spinal cord (SC) of 6-month-old GA-CFP mice compared to wildtype mice in the anterior horn of the spinal cord. No clear difference was observed for the astrocyte marker GFAP. *Scale bars* represent 100 µm. **c**, **d**, **f** Quantitative RT-PCR shows increased mRNA expression of CD68 and Iba1 but not GFAP in 6-month-old GA-CFP mice compared to 1-month-old GA-CFP mice and controls. Expression was normalized to GAPDH and β-actin using the ∆∆*Ct* method. *n*_(wt)_ = 3; *n*_(GA-CFP)_ = 3; Statistical analyses were performed by an unpaired *t* test (two-tailed; CD68_1-month_ *t* = 3.079, *df* = 4; Iba1_1-month_ *t* = 2.385, *df* = 4; GFAP_1-month_ *t* = 0.4147, *df* = 4; CD68_6-months_ *t* = 4.805, *df* = 4; Iba1_​6-months_ *t* = 6.399, *df* = 4; GFAP_​6-months_ *t* = 1.771, *df* = 4) and data are shown as mean ± SEM; *p < 0.05; **p < 0.01, *ns* not significant

Next, we analyzed the GA-CFP mice for signs of neuroinflammation. Immunohistochemistry for CD68 and Iba1 in 1- and 6-month-old mice revealed strong upregulation of these markers of phagocytic microglia in the spinal cord of 6-month-old GA-CFP mice (Fig. 4a, b) while little microglia activation was detectable at 1 month of age. Quantitative RT-PCR further confirmed enhanced mRNA expression of CD68 and Iba1 (Fig. 4c, d). In contrast, GFAP immunostaining and mRNA expression analysis revealed no signs of poly-GA-induced astrogliosis (Fig. 4e, f). Furthermore, in the neocortex of GA-CFP mice, a region lacking poly-GA pathology, no activation of CD68, Iba1 or GFAP was detected (Fig. S5a). Thus, neuronal poly-GA expression induces regional microglia activation in the absence of overt neuron loss in GA-CFP mice.

### GA-CFP mice develop progressive motor deficits

**Fig. 5:**
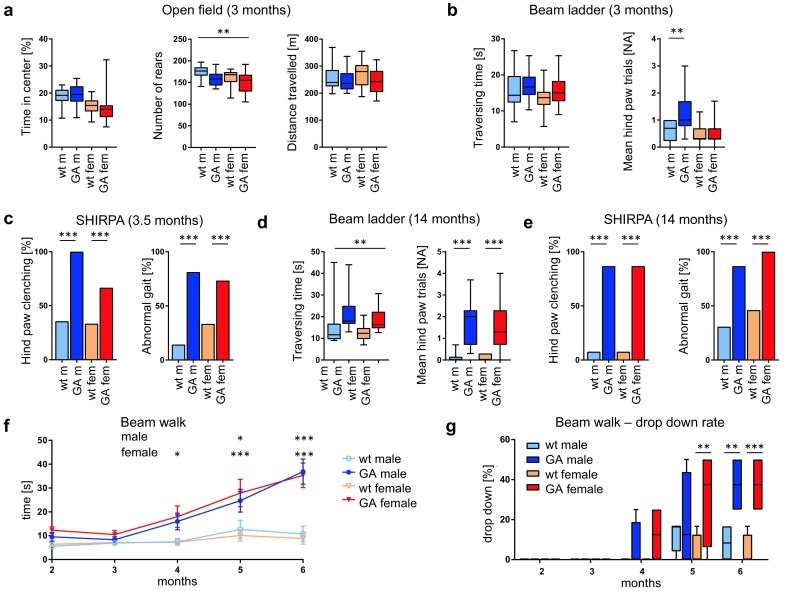
GA-CFP mice are less active and develop balancing deficits. **a**–**e** Neurological and behavioral analysis of GA-CFP and wildtype (wt) littermates at 3–4 months (**a**–**c**) and 14 months of age (**d**, **e**). **a** Open field analysis at 3 months. Automated analysis of the time spent in the center, the rearing activity (total count) and the total distance traveled within 20 min for the different genotypes and genders. **b** Beam ladder with irregular step distance. Automated analysis of average time needed to cross the ladder and the number of hind paw slips at the age of 4 months. **c** Gait analysis according to the SHIRPA protocol. Fraction of mice showing hindlimb clenching and abnormal gait is shown (at the age of 3.5 months). *n*_(GA-CFP male)_ = 16; *n*_(GA-CFP female)_ = 15; *n*_(wt male)_ = 14; *n*_(wt female)_ = 15 for all tests at 3 months of age. **d**, **e** Repetition of the beam ladder and SHIRPA analysis of GA-CFP and wildtype (wt) littermates at 14 months of age. *n*_(GA-CFP male)_ = 15; *n*_(GA-CFP female)_ = 15; *n*_(wt male)_ = 13; *n*_(wt female)_ = 13. **f**, **g** Longitudinal analysis using a balance beam. GA-CFP males and females take more time to cross the beam and fall more often than wildtype mice starting at 4 months. *n*_(GA-CFP male)_ = 4; *n*_(GA-CFP female)_ = 4; *n*_(wt male)_ = 6; *n*_(wt female)_ = 6. Statistical analysis of open field and beam ladder assay was performed by a two-way ANOVA between sex and genotype followed by Bonferroni post hoc test. Statistical analysis of the beam walk was performed by a two-way ANOVA between time and genotype for each sex followed by Bonferroni post hoc test. *Asterisks* for open field analysis and beam ladder traversing time depict significance of genotype effects [open field *F* (1, 56) = 7.579; beam ladder traversing time *F* (1, 52) = 10.2]. *Asterisks* for beam ladder hind paw trials, SHIRPA and beam walk depict significance of genotype and sex-dependent effects (Bonferroni). Statistical analysis of hind paw clenching and gait was performed by a Chi-square test. Data are shown as box plot with whiskers at the 1st and 99th percentille (**a**–**e**, ** g**) or as mean ± SEM (**f**); *p < 0.05; **p < 0.01; ***p < 0.001

To analyze the functional consequences of poly-GA pathology and its downstream effects, we performed in depth phenotyping of mice at 3–4 months of age when poly-GA pathology starts building up. Open field testing revealed no signs of anxiety as GA-CFP and wildtype mice spent a similar time in the center of the arena, but the decreased rearing activity of GA-CFP mice may indicate decreased motor performance or alterations in the relevant brain circuits (Fig. 5a). The overall distance traveled was not significantly reduced in GA-CFP mice. However, when walking across a beam ladder with irregular step distance, male GA-CFP mice showed significantly more hind paw slips, without a difference in total traversing time (Fig. 5b). In the SHIRPA analysis, the majority of GA-CFP mice showed hind paw clenching (84% compared to 35% of controls; Fig. 5c) and a broad, wagging gait (77% compared to 24% of controls). Grip strength of fore and hind limbs measured individually or combined was normal (data not shown). Decreased acoustic startle response and prepulse inhibition in GA-CFP mice suggest impaired sensorimotor gaiting and recruitment ability (Figs. S5b/c).

We repeated a subset of tests in 13–14-month-old mice. At this age, both male and female mice took significantly longer to cross the beam ladder and slipped more often with their hind paws (Fig. 5d). In the SHIRPA analysis, 87% of GA-CFP mice showed hind paw clenching compared to 8% of controls and abnormal gait (93% compared to 38% of controls) (Fig. 5e). However, out of the sixty mice used for this study, only three control mice and one GA-CFP mouse had died unexpectedly until the age of 16 months.

We additionally used an independent, smaller cohort of mice from 2–6 months of age for a longitudinal study focusing on memory function and motor coordination. While GA-CFP and wildtype mice initially gained weight normally for the first 6 months (Fig. S5d), transgenic mice showed reduced body weight compared to wildtype littermates after 15 months (male wildtype 38.6 g ± 3.1, male GA-CFP 32.1 g ± 1.7, female wildtype 31.1 g ± 4.0, female GA-CFP 26.4 g ± 2.3; ANOVA genotype effect *p* < 0.001). Hippocampus-dependent spatial memory of all mice was tested weekly using the Barnes maze; at all time-points, GA-CFP mice performed like their wildtype littermates, indicating that their spatial memory was not impaired (Fig. S5e). Moreover, we used the accelerated rotarod as a test for overall motor performance. Within 6 months, no difference in the performance of motor planning and physical condition was observed between GA-CFP mice and wildtype littermates (Fig. S5f). To measure balance and coordination more directly, mice were made to walk across a balance beam every week. The beam walk revealed progressive deficits in male and female transgenic mice (Fig. 5f, g). GA-CFP mice and their wildtype littermates showed similar performance from week 8 to 17, but from week 20 onward GA-CFP mice took a significantly longer time to cross the beam (Fig. 5f) or failed the test entirely by dropping down (Fig. 5g).

Taken together, GA-CFP mice develop progressive gait and balance impairments, while muscle strength and spatial memory are spared. These findings are consistent with the poly-GA pathology found exclusively in spinal cord, brainstem and cerebellum.

## Discussion

We generated the first germline transgenic mouse model of pure DPR pathology without (ggggcc)_n_ repeat RNA and analyzed the contribution of poly-GA to *C9orf72* ALS/FTD pathophysiology. We show that chronic accumulation of poly-GA proteins in the spinal cord, brain stem and deep cerebellar nuclei in GA-CFP mice results in progressive gait and balance impairment. These deficits are associated with the sequestration of p62, Rad23b and the chaperone-associated protein Mlf2. Accompanying regional microglia activation and modest TDP-43 phosphorylation suggest that poly-GA inclusions impair neuronal function prior to neuron loss in *C9orf72* ALS/FTD.

### GA-CFP mice model pure poly-GA pathology

The *Thy1*-driven GA-CFP mice express poly-GA proteins at levels roughly similar to human *C9orf72* ALS/FTD patients although with a different regional expression pattern. GA-CFP mice develop poly-GA pathology in motoneurons and other neurons of the spinal cord and brain stem and in deep cerebellar nuclei. In patients, poly-GA inclusions are found in the spinal cord, including motoneurons, but they are much more frequent in the neocortex, hippocampus, thalamus and cerebellum [20, 23, 31]. This has led to speculations that spinal cord motoneurons in patients may be particularly vulnerable to DPR protein expression. However, expression of poly-GA in GA-CFP mice does not cause a rapid loss of motoneurons. Our data indicate that poly-GA aggregates in neurons of the motor system disturb normal neuronal function in the absence of overt neuron loss, for example, by sequestration of cellular proteins.

We observed co-aggregation of poly-GA with p62 and Rad23 as reported previously [21, 23, 37]. For unknown reasons we did not detect sequestration of Unc119 into poly-GA inclusions in GA-CFP mice, which we had initially discovered in rat primary neurons and confirmed in *C9orf72* patients [21, 31]. Furthermore, we discovered that the chaperone-associated protein Mlf2 co-aggregates with half of the poly-GA inclusions in the spinal cord of GA-CFP mice, but only in 0.3–2.7% of the poly-GA inclusions in the cortex of *C9orf72* ALS/FTD patients. The differential co-aggregation of Mlf2 and Unc119 in GA-CFP mice and *C9orf72* cases highlights the importance of validating data from all model systems in patient tissue. The unexpected co-aggregation of Mlf2 and phospho-TDP-43 in *C9orf72* patients needs to be investigated further. While the drosophila homolog of Mlf2 has been shown to co-aggregate with poly-Q and modulate its toxicity in a Huntington’s disease model [16, 18], preliminary experiments in rat primary neuron culture did not show clear effects of Mlf2 on poly-GA toxicity (data not shown).

Consistent with cellular models [21, 38], poly-GA expression by itself did not induce visible TDP-43 inclusions. However, we did notice increased levels of phosphorylated TDP-43 in the urea-soluble fraction, indicating that poly-GA may contribute to the onset of TDP-43 pathology. Similarly, in an AAV-based model with much higher GFP-(GA)_50_ expression, only very few TDP-43 aggregates were detected [37]. While *C9orf72* patients show robust astrogliosis and microgliosis, the BAC transgenic and poly-GA expressing mice showed variable extent of astrocyte and microglia activation [15, 19, 26, 37]. The strong activation of microglia in the spinal cord of our GA-CFP mice may be due to neuronal dysfunction alarming microglia, extracellular release of poly-GA aggregates [35] or low levels of neurodegeneration not detected by our quantitative analysis. In ALS patients, microglia activation correlates with disease progression, and *C9orf72* carriers show higher microglia activation than non-carriers [4].

### Motor deficits in GA-CFP mice

So far, no transgenic *C9orf72* model has robustly replicated the full complement of ALS and/or FTD phenotypes in animals. BAC transgenic mice with human-like *C9orf72* expression levels show variable phenotypes and cannot differentiate between RNA and DPR toxicity [13]. Mice overexpressing poly-GA using AAV show signs of impaired nucleocytoplasmic transport and sequestration of Rad23b by poly-GA inclusions in the cortex [37]. Motor and balance deficits in these mice were attributed to the cerebellar poly-GA aggregation and neuron loss, because these mice rarely showed poly-GA inclusions in the spinal cord. In contrast, the motor system is directly affected in our transgenic GA-CFP mice. The motor phenotype of GA-CFP mice is consistent with the predominant expression of poly-GA in spinal cord and brain stem. Poly-GA inclusions in the deep cerebellar nuclei may further contribute to this phenotype. The impaired acoustic startle response and its prepulse inhibition, together with the distribution of poly-GA inclusions in the brainstem, suggest that poly-GA inhibits activity of this complex circuit involving the brain stem [8, 36]. However, we cannot retrospectively exclude that early deafness may have affected these measurement in GA-CFP mice, because C57BL/6 mice commonly develop hearing loss at 3–6 months. By 14 months all female mice were deaf, while male GA-CFP mice were more severely affected than controls (data not shown).

The most prominent phenotype of GA-CFP mice, however, is their impaired balance and gait, which particularly affected the hind limbs. This is consistent with the widespread poly-GA pathology in the lumbar segments both in motoneurons and laminae IV, V and VI neurons implicated in proprioception. At 4 months of age, male mice slip more often with their hind paws on the beam ladder and show decreased rearing activity in the open field arena indicating deficits in motor control, which requires cortical input and proper function of the spinal cord circuits. Both male and female GA-CFP mice show enhanced hind limb clenching and an abnormal gait as well as progressive impairment on the balance beam starting at 3–4 months of age. Normal grip strength and endurance in the rotarod corroborates the absence of overt motoneuron loss and suggests poly-GA disturbs proper neuronal function and thus impairs coordination and motor control.

### Summary

In patients, expression of poly-GA and the other DPR species correlates poorly with regional neuron loss [20, 31] and DPR pathology precedes overt ALS/FTD symptoms by many years [2, 27]. Our findings suggest that poly-GA-induced protein sequestration and regional microglia activation may be responsible for the prodromal cognitive deficits observed prior to complete ALS/FTD symptoms in *C9orf72* mutation carriers [29]. Combined chronic DPR and RNA toxicity likely trigger the second disease stage with TDP-43 pathology and overt neuron loss [7]. Finally, GA-CFP mice are a good model to test poly-GA-based therapeutic approaches, because motor deficits appear early and homogeneously in all animals.

## Electronic supplementary material

Below is the link to the electronic supplementary material.

Supplementary material 1 (PDF 1410 kb)
