# Supplementary material for: Spinal poly-GA inclusions in a C9orf72 mouse model trigger motor deficits and inflammation without neuron loss
Source: Acta Neuropathol. 2017 Apr 13;134(2):241–54. doi: 10.1007/s00401-017-1711-0 (PMC5508040; doi:10.1007/s00401-017-1711-0)
Supplement: Supplementary file 1 — Supplementary material 1 (PDF 1410 kb) [file 401_2017_1711_MOESM1_ESM.pdf]

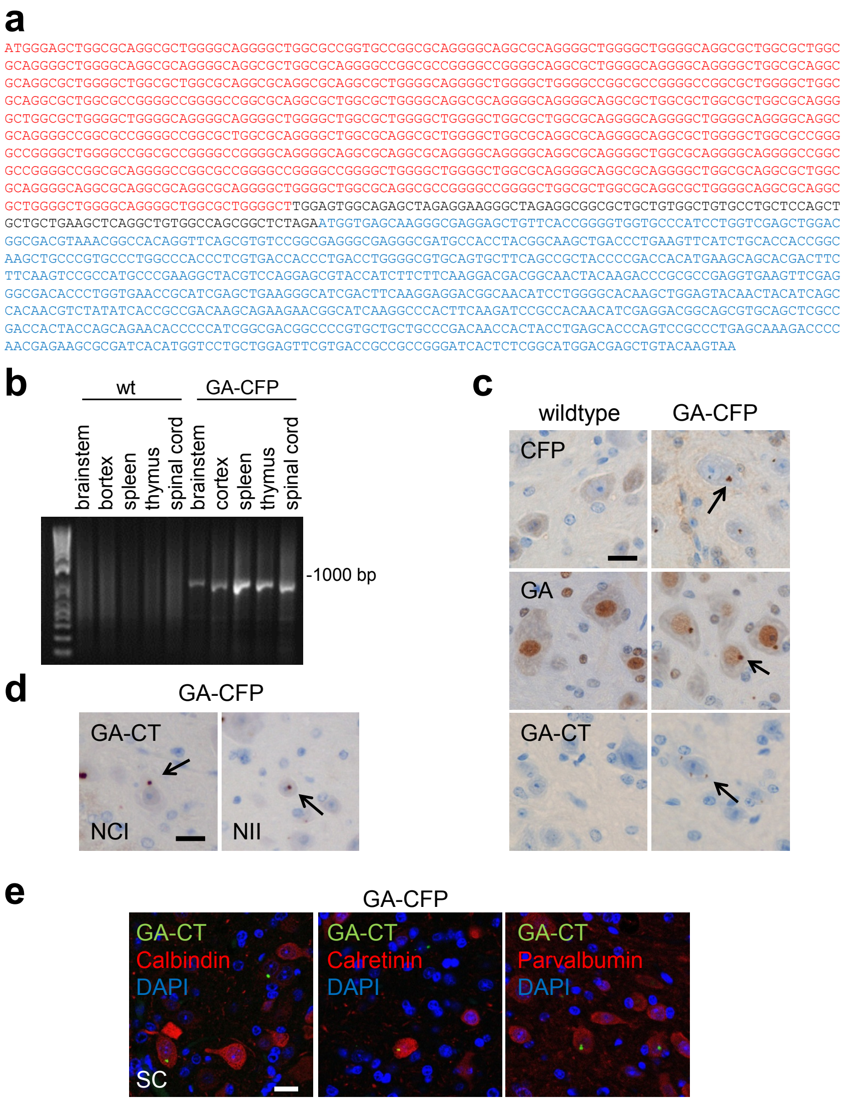

Figure S1: Transgenic mice show germline transmission of a GA-CFP construct

(a) Coding sequence of the transgene expressing $(GA)_{149}$-CFP. The poly-GA sequence with strongly reduced ggggcc content (red), the C-terminal tail (CT) expressed from the endogenous *C9orf72* repeat locus in the poly-GA reading frame (black) and CFP (blue). **(b)** In GA-CFP transgenic mice the full length poly-GA sequence is detectable in genomic DNA extracted from brainstem, cortex, spleen, thymus and spinal cord. **(c)** Immunohistochemical staining of the spinal cord for CFP, GA (5F2-HRP) and the C-terminal tail GA-CT (5C3) in GA-CFP mice (right column) and wildtype mice (left column) at 6 months of age. **(d)** Poly-GA forms predominantly neuronal cytoplasmic inclusions (NCI) and rarely neuronal intranuclear inclusions (NII). **(e)** Double immunofluorescence staining shows poly-GA inclusions in calbindin, calretinin and parvalbumin positive neurons in the spinal cord (SC). Scale bars represent 20 µm.

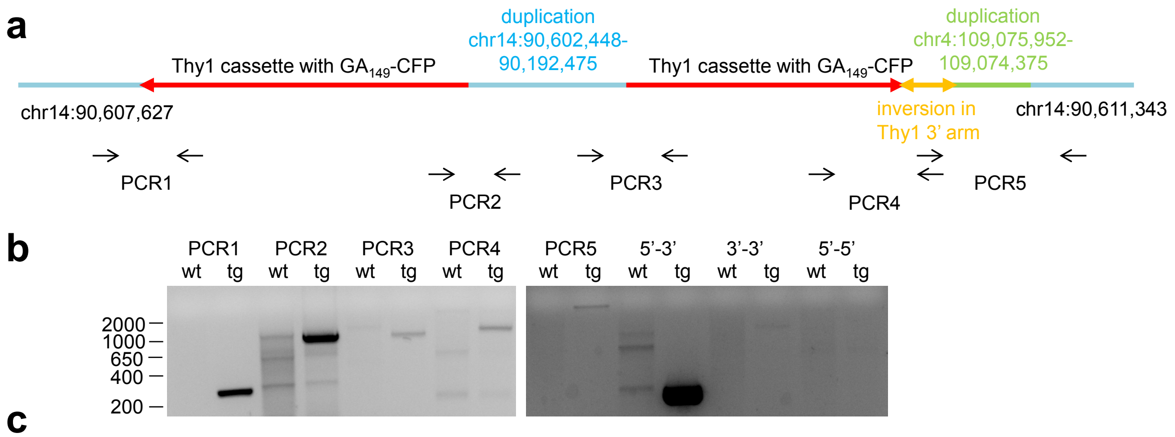

c

```
PCR1                                  <<< chr9:44,049,192 Thy 3' arm
…CCCACTGATGTCCAACAAAGCCATCCTCTGCAACAAACCCAGAACGGCCTGGGGTA…
            chr14:90,607,627 >>>

PCR2&PCR3                         <<< chr14:90,602,448    chr14:90,192,475 >>>
…AAATGAAATTTAAAATTGTTTATACTATTTGTGTGCAGTGATTAGAGCCT…ACAGATTCCATGCAATCCCCATGCACTGTTTCCCTAACAGGGC…
      Thy1 5'arm chr9:44,041,999 >>>                                      <<< chr9:44,042,823

PCR4                 <<<vector backbone                      <<<chr9:44,050,120 Thy1 inversion
…TTCTCTCTGCTGAGTCATCTGGCGATCGGTGCGGGCCTCTTCGCTATTACGCCTGGATCAATTCTAGGACTTAAGGTAGGAAACTAAG…
Thy 3'  chr9:44,048,846>>>

PCR5                 <<< chr4:109,075,952          chr4:109,074,375 >>>
…GCAAATGGATCCCATTACAACCCAGTATTAGCGATCACTCTAAAT…GCACATGCCATTGTTCACGTGTCTTAAAATCTTACCAACAAGAAATCT…
 Thy1 inv  chr9:44,049,191 >>>                                       <<< chr14:90,611,343
```

Figure S2: Thy1 cassette is integrated at an intergenic site on chromosome 14

(**A**) Schema of the transgene integration on chromosome 14. Whole genome sequencing at ~100x coverage revealed integration of two blocks of tandem repeats of the transgene integrated in chromosome 14 at position 90,607,627-90,611,343 leading to a 3716bp intergenic deletion about 330 kb downstream of the nearest transcript a long non-coding RNA (4930474H20Rik). The integration involves duplication of a 410kb stretch of chromosome 14 (including the 3' part of 4930474H20Rik without the promoter region) and a 1577 nucleotide stretch from chromosome 4 (intronic region of Ospbl9). All genome coordinates are based on the GRC38/mm10 mouse genome assembly. Coordinates for the Thy1 cassette correspond to the genomic numbering of the endogenous mouse Thy1 locus on chromosome 9, spanning chr9:44,041,811-44,045,981 (5' arm) and chr9:44,047,482-44,050,120 (3'arm) in the transgene vector. The increased sequence coverage in that region suggests that at least 10 copies of the transgene are integrated in the block. The 3' arm of the distal Thy1 cassette fused to the chromosome 4 duplication contains a 969 nucleotide inversion. Arrows indicate primers used for PCR validation. Primer sequences PCR1 (cctcccaccacctcaatgag, tgggctggagtacgaaacat), PCR2 (aggctctaatcactgcacaca, cctggcttgtttctgcttcc), PCR3 (acccatgatattttcctagatgca, cctggcttgtttctgcttcc), PCR4 (aattaccaccactcgctccc, attcccttgcctcctgtctc),

PCR5 (tcatctgttgtgtaaaaggcca, gggagcgagtggtggtaatt). Not drawn to scale. (**B**) Genomic PCR from wildtype and transgenic littermates confirms integration and chromosomal rearrangement. Bands specific to GA-CFP mice were excised and sequenced. To analyze tandem inserts we additionally used primers facing outside of the Thy1 cassette (5'-3' tcgttcactgtccttattctctctc, gtcaggcttgctggtagg; 3'-3' tcgttcactgtccttattctctctc; 5'-5' gtcaggcttgctggtagg) confirming the presence of head to tail tandem repeats. Other tandem orientations containing larger 5’ or 3’ deletions of the transgene cassette may exist. (**C**) Sequence at the chromosomal rearrangements involving the Thy1 cassette based on Sanger sequencing of the PCR products from (B).

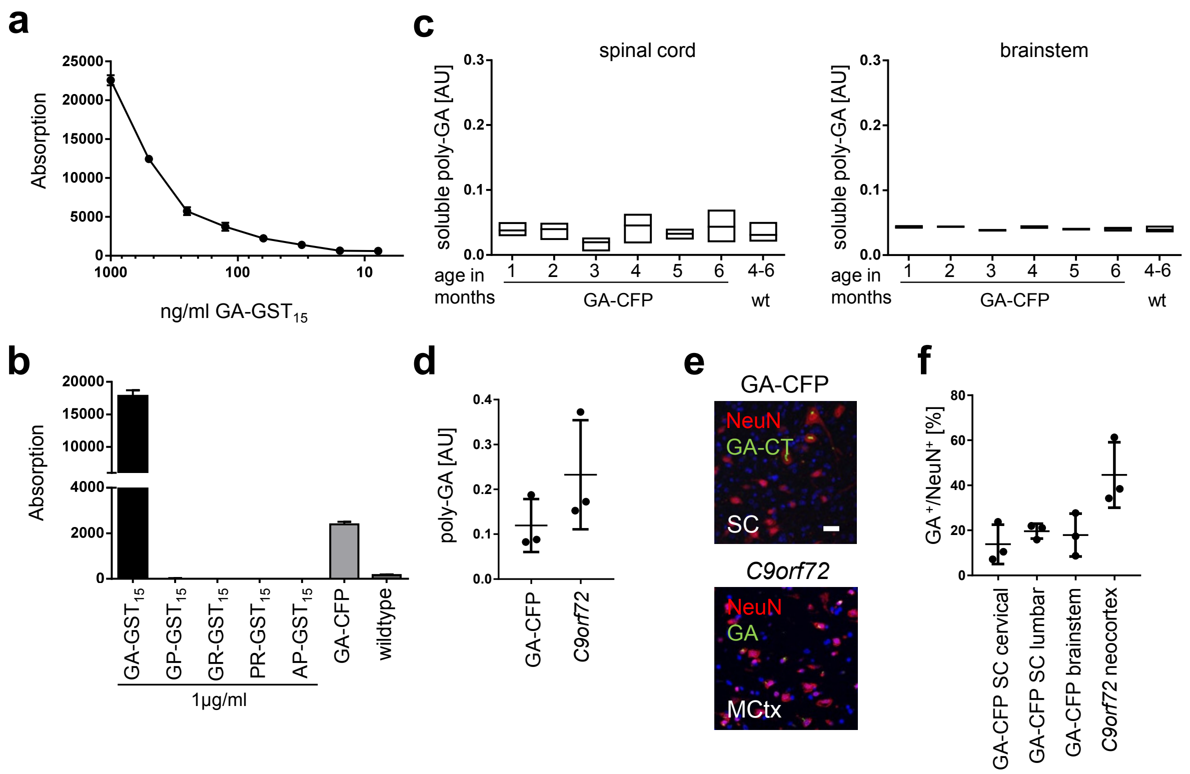

Figure S3: Comparison of poly-GA in GA-CFP spinal cord and *C9orf72* ALS/FTD patient neocortex

(a) Poly-GA sandwich immunoassay using biotinylated and sulfo-tagged α-GA 5F2 antibody shows the detection limit on recombinant GST-GA_{15}. **(b)** Anti-GA immunoassay specifically detects GST-GA_{15} and RIPA-insoluble poly-GA in the spinal cord of a GA-CFP mouse. No signal was detected for other DPR antigens or in wildtype mice. Mean + SEM. **(c)** poly-GA immunoassay from the RIPA-soluble fraction of spinal cord (SC) and brainstem (BS) of 1 to 6 months old GA-CFP mice (n = 3 mice per time-point; measured in duplicates, box plot shows minimum, mean and maximum) detects no poly-GA signal above background. Compare data from RIPA-insoluble fraction in Figs. 2d/e. AU = arbitrary unit. **(d)** Poly-GA protein levels in the RIPA-insoluble fraction of the spinal cord of 4-6 months-old GA-CFP mice are not grossly higher than in the motor cortex of *C9orf72* mutation carriers. All tissues were analyzed in parallel following the same extraction protocol. Mean, minimum and maximum are shown. Due to the high variability of poly-GA pathology in *C9orf72* patients and the small cohort size we did not perform statistical analysis. Mean ± SD. **(e, f)** Double immunofluorescence with the neuronal marker NeuN shows that neuronal poly-GA inclusions are less common in the spinal cord and brainstem of 6-months-old GA-CFP mice than in the motor cortex of *C9orf72* patients. $n_{GA\text{-}CFP} = 3$; $n_{C9orf72} = 3$; For quantification 3-4 images (424.7 x 424.7 μm^2) were taken and the fraction of poly-GA inclusion bearing NeuN positive neurons were counted. Mean ± SD. AU=arbitrary unit. Scale bar represents 20 µm.

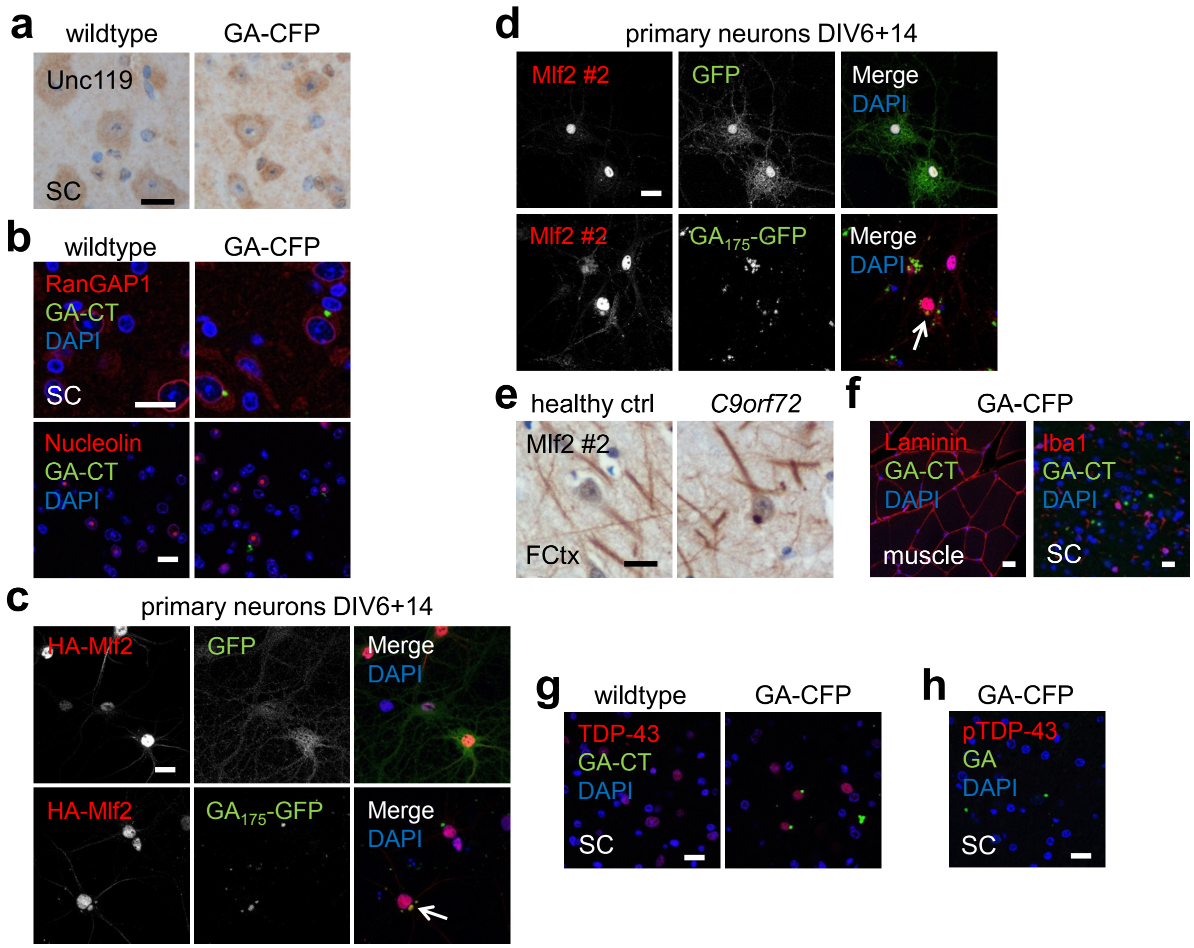

Figure S4: poly-GA does not affect TDP-43, RanGAP1 and nucleolin but is co-localized with Mlf2

(a) Immunohistochemistry did not reveal Unc119 aggregates in the spinal cord (SC) of GA-CFP mice. **(b)** Immunofluorescence in 12 month-old GA-CFP mice shows similar RanGAP1 and nucleolin localization in GA-CFP mice and wildtype littermates. **(c)** Lentiviral co-transduction of HA-Mlf2 with either GA_{175}-GFP or GFP control in primary hippocampal neurons of wildtype rats at 6 days *in vitro*. Immunofluorescence 14 days later (DIV6+14) detects specific co-localization of HA-Mlf2 with GA_{175}-GFP. **(d)** Mlf2 immunostaining in rat primary hippocampal transduced with GA_{175}-GFP or GFP detects specific co-localization of endogenous Mlf2 with GA_{175}-GFP (arrow). **(e)** Immunohistochemistry using a second Mlf2 antibody (compare Figs. 2c,d) detects specific Mlf2 aggregates in the frontal cortex (FCtx) of *C9orf72* ALS/FTLD patients. No inclusions were detectable in healthy controls. Scale bar represents 20 µm. **(f)** Immunofluorescence staining showed no poly-GA aggregates in laminin-labeled muscle fibers (quadriceps femoris, left) or Iba1-positive microglial cells (spinal cord, right). **(g, h)** Immunofluorescence analysis did not reveal cytoplasmic mislocalisation of TDP-43 or phosphorylation at serine 409/410 in GA-CFP mice. Scale bars represent 20µm.

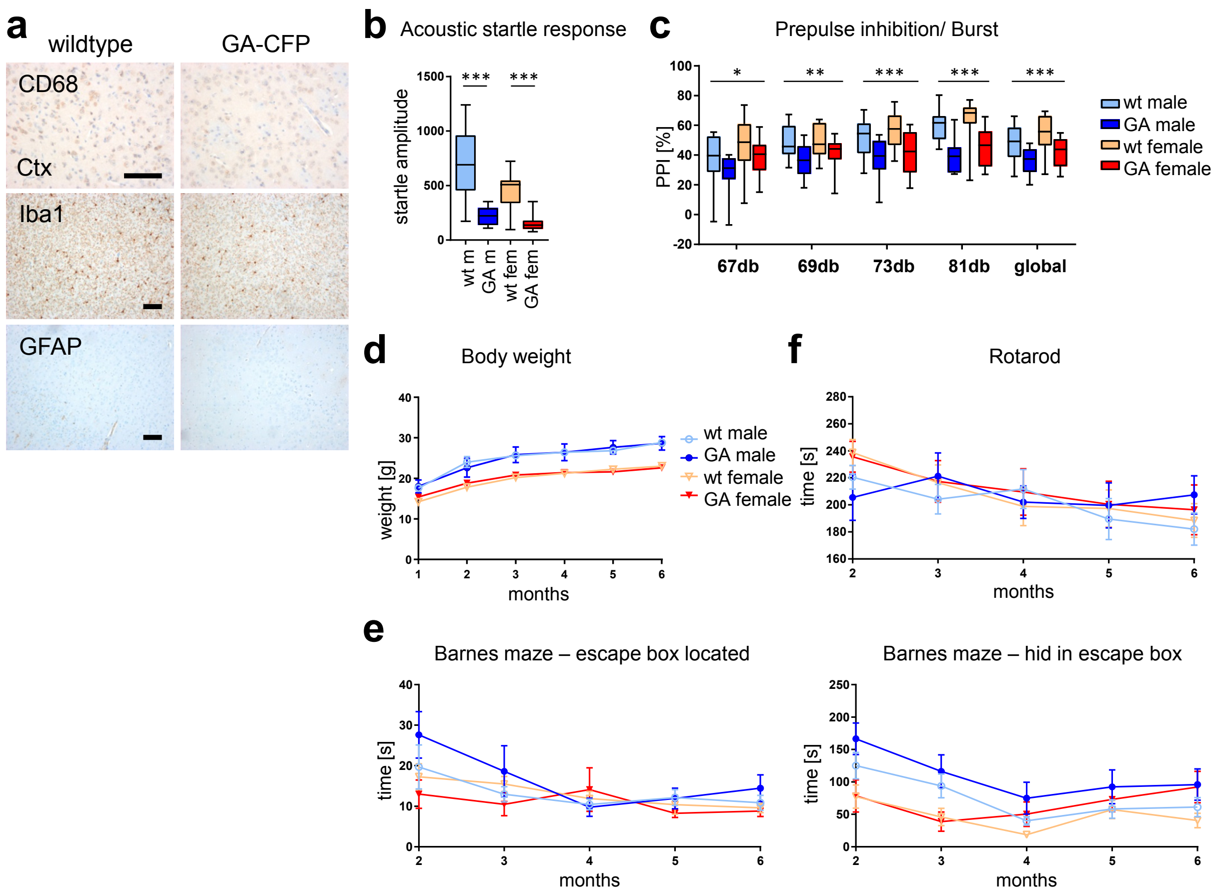

Figure S5: GA-CFP mice show no microglia activation in the neocortex, normal spatial memory, but impaired prepulse inhibition

(a) Immunohistochemistry of neocortex shows comparable levels of CD68, Iba1 and GFAP in GA-CFP and control mice at 6 months of age. Scale bars represent 100µm. **(b, c)** Neurological analysis of GA-CFP and wildtype littermates at 3-4 months of age. Box plot with whiskers at 1st and 99th percentile. $n_{(GA\text{-}CFP\ male)}$ = 16; $n_{(GA\text{-}CFP\ female)}$ = 15; $n_{(wt\ male)}$ = 14; $n_{(wt\ female)}$ = 15. Acoustic startle response **(b)** and its prepulse inhibition **(c)** are decreased in GA-CFP male and female mice at 13 weeks. Prepulse inhibition 67db F (1, 56) = 4.588; 69db F (1, 56) = 10.52; 73db F (1, 56) = 18.35; 81db F (1, 56) = 40.56; global db F (1, 56) = 21.39). Asterisks for prepulse inhibition depict significance of genotype, asterisks for acoustic startle depict significance of genotype and sex-dependent effects (Bonferroni). **(d-f)** Longitudinal analysis of body weight (**d**), spatial memory (**e**) and motor performance (**f**) of GA-CFP mice and wildtype littermates. **(d)** No differences in the body weight were observed between transgenic and wildtype male and female mice from birth until the age of 6 months. **(e)** Spatial learning and long-term memory was normal in 2-6 months old transgenic or wildtype mice. Left panel shows the time needed to locate the escape box, the

right panel shows the time until the mice crawl into the escape box. **(f)** No differences in the rotarod performance of GA-CFP mice up to 6 month compared to wildtype mice. Statistical analysis of the body weight and Barnes maze was performed by a 2-way ANOVA between time and genotype for each sex followed by Bonferroni post hoc test. $n_{(GA\text{-}CFP\ male)} = 4$; $n_{(GA\text{-}CFP\ female)} = 4$; $n_{(wt\ male)} = 6$; $n_{(wt\ female)} = 6$. Data are shown as mean ± SEM; *$p < 0.05$; **$p < 0.01$; ***$p < 0.001$.

Table S1: Colocalization of poly-GA inclusions in GA-CFP mice

Quantitative analysis of double immunofluorescence stainings from GA-CFP mice to address colocalization of poly-GA with p62 and RanGAP1 and mislocalization of total TDP-43 and TDP-43 phosphorylated at Ser409/410. Stainings were performed on spinal cord slices from three 12-month-old mice. Mean percentages ± SD are shown.

Protein	counted GA aggregates	Mean ± SD
p62	110	94.55 ± 5.13 % colocalization
RanGAP1	112	0.00 % colocalization or mislocalization
TDP-43	123	0.00 % mislocalization
pTDP-43	117	0.00 % mislocalization

Table S2: Colocalization of Mlf2 and poly-GA in *C9orf72* patients and GA-CFP mice

Double immunostainings of Mlf2 and poly-GA in C9orf72 patients and GA-CFP mice. The fraction of poly-GA inclusions containing Mlf2 was manually quantified in the indicated regions. Three *C9orf72* patients and three GA-CFP mice (12-month-old) were analyzed. Mean percentages ± SD are shown.

Region	counted GA aggregates	% Mlf2
C9orf72 FCtx	283	1.06 ± 1.08 %
C9orf72 MCtx	162	0.62 ± 0.80 %
C9orf72 OCtx	338	0.30 ± 0.35 %
C9orf72 DG	677	2.66 ± 1.45 %
GA-CFP SC	116	55.17 ± 5.32 %
